# Protection from Lethal Clostridioides difficile Infection via Intraspecies Competition for Cogerminant

**DOI:** 10.1128/mBio.00522-21

**Published:** 2021-03-30

**Authors:** Jhansi L. Leslie, Matthew L. Jenior, Kimberly C. Vendrov, Alexandra K. Standke, Madeline R. Barron, Tricia J. O’Brien, Lavinia Unverdorben, Pariyamon Thaprawat, Ingrid L. Bergin, Patrick D. Schloss, Vincent B. Young

**Affiliations:** a Department of Microbiology and Immunology, University of Michigan Medical School, Ann Arbor, Michigan, USA; b Division of Infectious Diseases, Department of Internal Medicine, University of Michigan Medical School, Ann Arbor, Michigan, USA; c The Unit for Laboratory Animal Medicine, University of Michigan, Ann Arbor, Michigan, USA; University of Oklahoma Health Sciences Center

**Keywords:** *Clostridioides difficile*, amino acids, competition, germination, murine model

## Abstract

Clostridioides difficile, a Gram-positive, spore-forming bacterium, is the primary cause of infectious nosocomial diarrhea. Antibiotics are a major risk factor for C. difficile infection (CDI), as they disrupt the gut microbial community, enabling increased germination of spores and growth of vegetative C. difficile. To date, the only single-species bacterial preparation that has demonstrated efficacy in reducing recurrent CDI in humans is nontoxigenic C. difficile. Using multiple infection models, we determined that precolonization with a less virulent strain is sufficient to protect from challenge with a lethal strain of C. difficile, surprisingly even in the absence of adaptive immunity. Additionally, we showed that protection is dependent on high levels of colonization by the less virulent strain and that it is mediated by exclusion of the invading strain. Our results suggest that reduction of amino acids, specifically glycine following colonization by the first strain of C. difficile, is sufficient to decrease germination of the second strain, thereby limiting colonization by the lethal strain.

## Abstract (precis)

Antibiotic-associated colitis is often caused by infection with the bacterium Clostridioides difficile. In this study, we found that reduction of the amino acid glycine by precolonization with a less virulent strain of C. difficile is sufficient to decrease germination of a second strain.

## INTRODUCTION

Clostridioides difficile, a Gram-positive, spore-forming bacterium, is the primary cause of infectious nosocomial diarrhea (1). Susceptibility to C. difficile infection (CDI) results from perturbations of the gut microbial community, enabling increased germination of spores and growth of vegetative C. difficile (2, 3). Following colonization, vegetative C. difficile produces the toxins TcdA and TcdB, which are glycosyltransferases that inactivate cellular GTPases (4). Inactivation of these key cellular proteins results in damage of the colonic epithelium and inflammation manifesting as colitis, diarrhea, and, in severe cases, toxic megacolon or even death. Currently, the principal treatment for acute CDI is the antibiotic vancomycin (5). While this treatment limits vegetative C. difficile, this therapy further disrupts the gut microbiota, delaying community recovery, potentially leading to recurrent disease (6).

Due to the high rate of recurrent infection associated with existing treatment for CDI, alternative approaches that spare or even restore the gut microbiota have been a focus of recent work. As is the case with many toxin-mediated diseases, early studies noted that generation of a humoral immune response to the toxins could be sufficient to protect against disease (7). A recent clinical trial demonstrated that patients receiving monoclonal antibodies targeting the toxins were 50% less likely to experience recurrent disease (8, 9). Unlike antibiotics, antibody therapy prevents illness while likely sparing the microbial community. Recently, a nontoxigenic strain of C. difficile was demonstrated to successfully reduce the rate of recurrent CDI by approximately 50% (10, 11). The prevailing hypothesis is that the protection provided by the nontoxigenic strain is mediated by competitive exclusion, limiting the ability of toxigenic C. difficile to colonize the gut (12). However, this has never been conclusively demonstrated. Using a murine model of CDI, we sought to address this question.

We report here that, in the absence of adaptive immunity, precolonization with one strain of C. difficile is sufficient to provide protection from lethal infection with another. Using gnotobiotic mice, we show that protection is mediated by limiting colonization of the highly virulent strain. Furthermore, we provide evidence that exclusion is not predicated on nutrient-based limitation of the vegetative form of the invading strain but, rather, on depletion of the cogerminant glycine. This work is important, as it is the first study to identify a possible mechanism through which precolonization with C. difficile, a current clinical therapy, provides protection from recurrent CDI. Furthermore, limitation of germination due to decreased levels of glycine in the gut is a novel paradigm for colonization resistance.

## RESULTS

### Development of a murine model of persistent C. difficile colonization.

**FIG 1:**
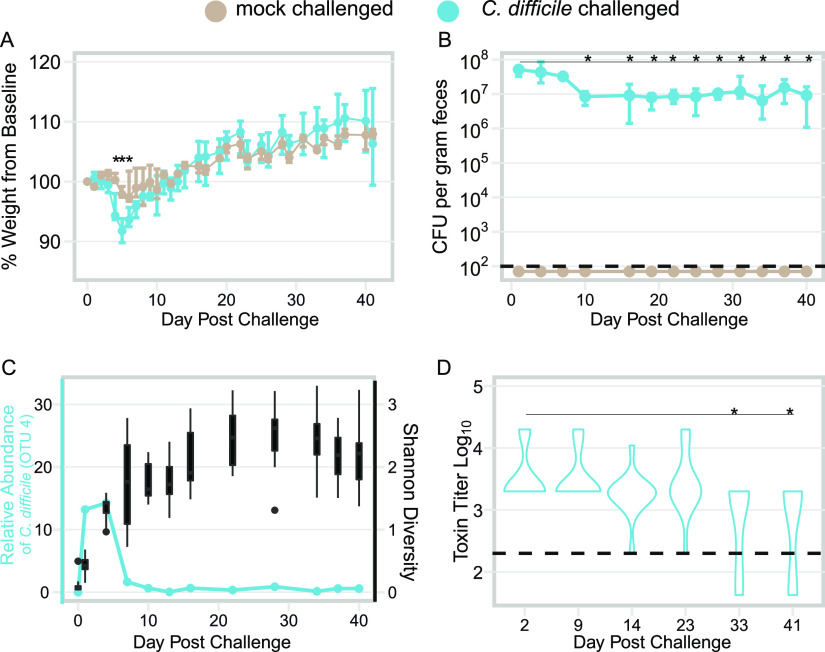
Murine model of persistent C. difficile colonization. (A) Change in weight relative to day of infection in infected and mock-challenged mice. Points represent median weight; bars are the upper and lower quartiles. Infected mice are colored blue (*n* = 14), while data from mock-infected animals are shown in tan (*n* = 8). Following correction for multiple comparisons, weight loss in infected mice was only significantly different than mock-challenged mice on days 4, 5, and 6 postinfection; *P < *0.05. (B) C. difficile colonization over time as determined by quantitative culture. Colonization significantly decreased and remained significantly lower by day 10 postinfection relative to day 1 postinfection 40 (*n* = 14); *P < *0.01. The dashed line represents the limit of detection of 100 CFU/g feces. (C) Relative abundance of OTU 4 (C. difficile) over the course of the experiment (blue line) is plotted on the left axis, while Shannon diversity of the infected mice over the course of the experiment is plotted on the right axis (black box plots). (D) Fecal toxin activity remains detectable throughout the experiment. Toxin titers on day 33 and day 40 are significantly different from day 1 postinfection levels (*n* = 14); *P < *0.05. Statistical significance for all data was calculated using a Wilcoxon test with Benjamin-Hochberg correction. The dashed line represents the limit of detection (LOD) for each assay; for visual clarity, samples that were below the limit of detection were plotted below the line. However, for statistical analysis, the value of $\text{LOD/}\sqrt{2}$ was substituted for undetected values.

The only single-species bacterial preparation that has demonstrated efficacy in reducing recurrent CDI in humans is nontoxigenic C. difficile (10). However, the mechanisms by which one strain of C. difficile prevents colonization of another are currently unknown. To begin to address this question, we developed a model of persistent C. difficile colonization. Mice were made susceptible to colonization via administration of the antibiotic cefoperazone. Following 2 days off the antibiotic, mice were either mock challenged or challenged with C. difficile strain (str.) 630 spores. Compared to mock-challenged animals, infected animals displayed significant weight loss between days 4 to 6 postchallenge (Fig. 1A; *P < *0.05). After 10 days, infected mice remained stably colonized at approximately 10^7^ CFU/g feces despite a significant reduction in fecal levels of C. difficile relative to levels on day 1 postinfection (Fig. 1B; *P < *0.01). Coincident with the decrease in colonization, infected animals recovered weight until they were indistinguishable from the mock-infected controls (Fig. 1A). Additionally, the diversity of the gut microbiota increased within the first 10 days of infection, corresponding to the recovery of weight and decrease in pathogen colonization (Fig. 1C, box plots, right axis). Throughout the experiment, toxin was detected in the feces of most of the infected mice. However, toxin activity started to decrease by day 14 and was significantly decreased by day 33 postchallenge relative to early in the infection (Fig. 1D). Together, these data demonstrate that C. difficile str. 630 can persistently colonize wild-type mice as a minority member of the gut microbiota (Fig. 1C, left axis).

### Precolonization with C. difficile protects mice from challenge with a highly virulent strain even in the absence of adaptive immune responses.

**FIG 2:**
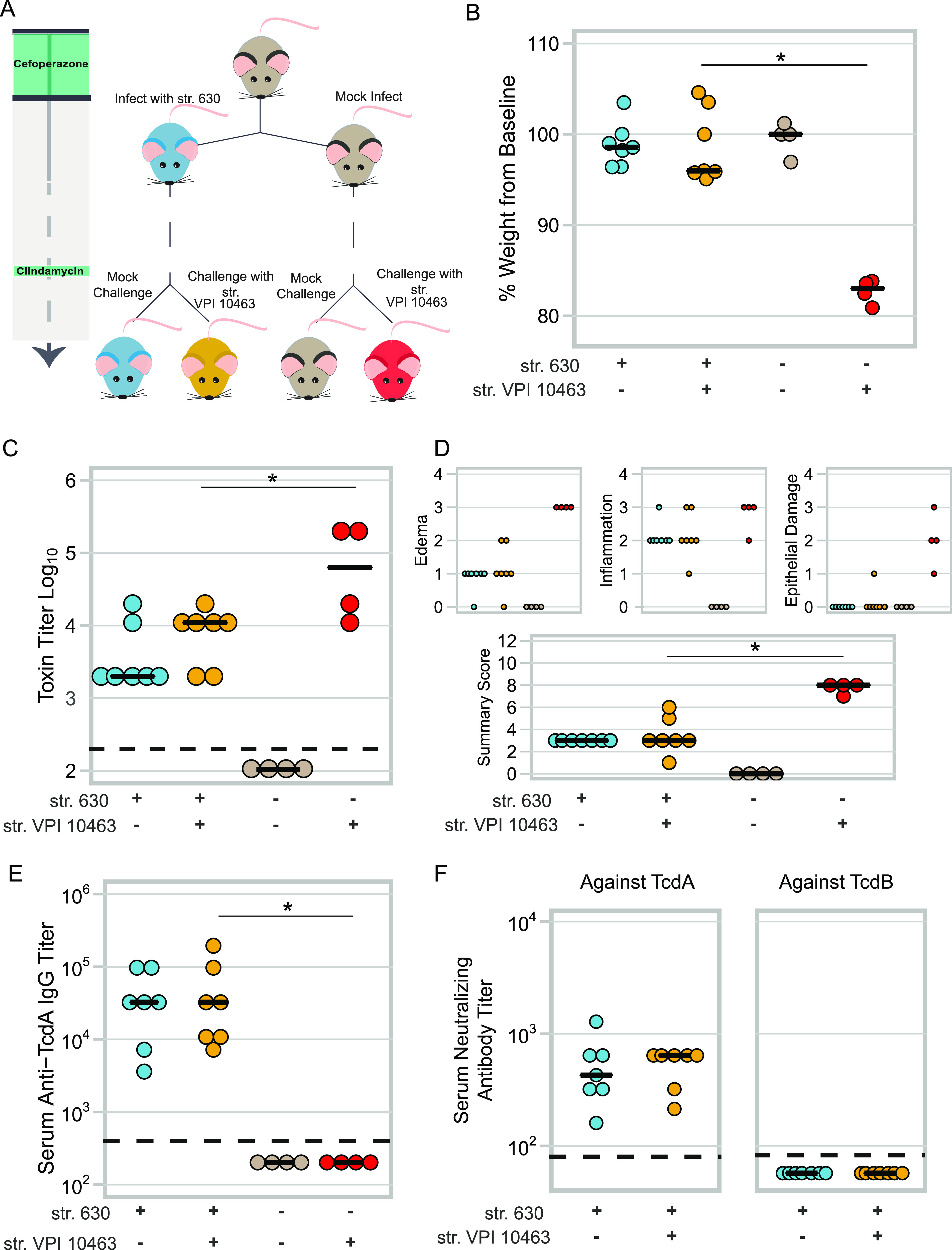
Mice precolonized with C. difficile str. 630 are protected from challenge with a lethal strain. (A) Schematic of experimental conditions. Colors corresponding to treatment groups are carried throughout the figure. (B) Change in weight at time of necropsy relative to weight on day of challenge, day 44 of the model. Mice colonized with C. difficile str. 630 and challenged with the lethal strain (VPI 10463) (*n* = 4) are protected from weight loss, whereas mice that had no exposure to C. difficile str. 630 experienced significant weight loss (*n* = 4); *P < *0.01. (C) Toxin titer from intestinal content from mice in panel A as measured by Vero cell-rounding assay. Mice colonized with C. difficile str. 630 and then challenged with C. difficile str. VPI 10463 have a lower toxin titer than naive mice challenged with str. VPI 10463; *P < *0.05. (D) Histopathology from colons of the mice in panel A. Small panels depict scores for each component of the summary score. Summary score of str. VPI 10463-challenged and str. 630-colonized versus str. VPI 10463-challenged naive mice, *P < *0.01. (E) Titer of serum IgG against TcdA at conclusion of experiment as measured by ELISA. Limit of detection was a titer of 400; *P < *0.01. (F) Neutralizing titer of serum against TcdA or TcdB. For all data, statistical significance between the C. difficile str. VPI 10463-challenged, str. 630-colonized and str. VPI 10463-challenged naive mice was determined by Wilcoxon test. The dashed line represents the limit of detection for each assay; for visual clarity, samples that were below the limit of detection were plotted below the line.

To determine if a resident strain of C. difficile protects mice from challenge with a second strain in the context of perturbation to the gut microbiota, we administered a second antibiotic, clindamycin, to both the colonized and uncolonized mice. Previous work from our group demonstrated that a single dose of clindamycin rendered mice susceptible to colonization following 6 weeks of recovery from cefoperazone treatment (13). Clindamycin did not result in weight loss in either group of mice (Fig. S1A in the supplemental material). However, levels of str. 630 in the colonized mice significantly increased following administration of clindamycin, likely because it is resistant to the antibiotic (Fig. S1B; *P* < 0.001) (14). The day after mice were given clindamycin, they were challenged with 10^5^ CFU of str. VPI 10463 spores. Strain VPI 10463 is lethal in this model of infection (13). To control for possible variations in the microbiota across cages, mice from str. 630-infected and mock-infected (naive) cages were split into two groups (Fig. 2A). Approximately half the mice in a cage were challenged with str. VPI 10463, while the remaining mice were placed in a new cage and received mock infection. Naive mice that had neither been infected with str. 630 nor str. VPI 10463 maintained stable weight; however, naive mice that were challenged with str. VPI 10463 lost a significant amount of weight and had to be euthanized (Fig. 2B; *P < *0.01). Mice that were persistently colonized with str. 630 did not lose weight despite being challenged with the lethal strain. In addition, str. 630-precolonized mice had significantly lower toxin titers than the naive mice challenged with str. VPI 10463 (Fig. 2C; *P < *0.05). This finding was confirmed by histopathology, as the summary scores of the histopathological damage in the colon were significantly less in the str. 630-colonized mice challenged with str. VPI 10463 than the naive mice challenged with str. VPI 10463 (Fig. 2D; *P < *0.01).

10.1128/mBio.00522-21.1 FIG S1 Effect of clindamycin on weight and colonization levels. (A) Change in weight from the day mice were given clindamycin to the following day. Mock-infected mice (or naive animals) are a reference point. There was not a significant difference between the infected or mock-infected mice following administration of clindamycin; *P > *0.05. (B) C. difficile colonization in infected animals 1 day prior to administration of clindamycin and 1 day following. Clindamycin significantly increases levels of C. difficile str. 630 in colonized mice; *P < *0.001. For the data included in each figure, statistical significance was calculated using a Wilcoxon test. Download FIG S1, EPS file, 1.5 MB. Copyright © 2021 Leslie et al. 2021 Leslie et al. https://creativecommons.org/licenses/by/4.0/ This content is distributed under the terms of the Creative Commons Attribution 4.0 International license.

Since both strains express nearly identical forms of TcdA and TcdB and we observed decreased signs of disease in the primary infection after a week of first being challenged, we questioned if protection might be due to the development of a humoral immune response to the toxins (7, 15). We found that mice previously infected with str. 630 developed a high anti-TcdA titer with a median titer of 1:32,400, with a portion of these antibodies being neutralizing, capable of preventing TcdA-mediated cell rounding (Fig. 2E, str. 630-colonized versus naive mice, *P < *0.01; Fig. 2F).

**FIG 3:**
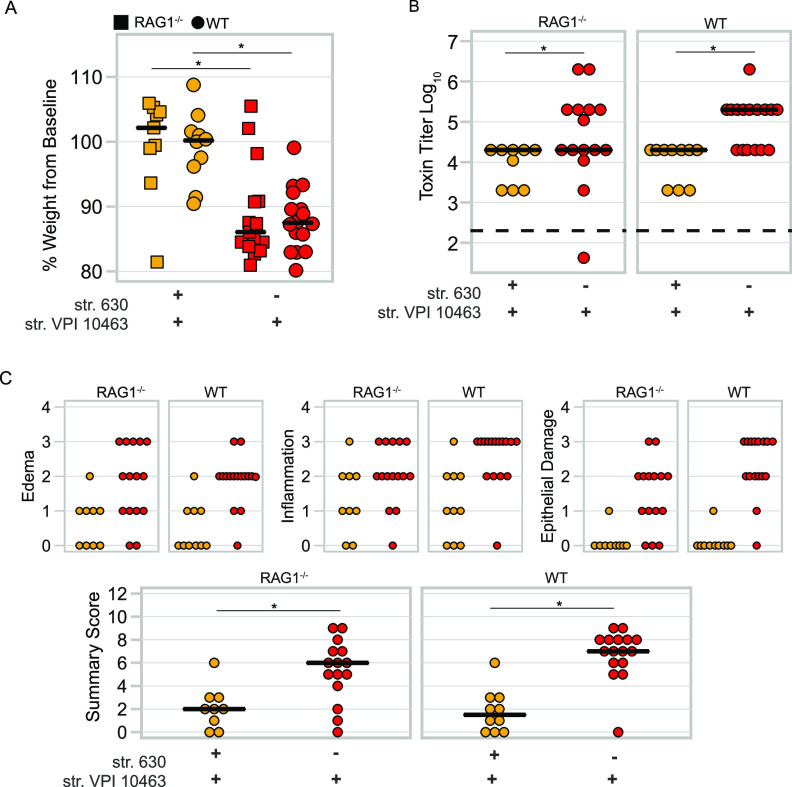
RAG1^−/−^ mice precolonized with C. difficile are protected from challenge with a lethal strain of C. difficile. (A) Change in weight at time of necropsy relative to weight on day of challenge, day 44 of the model. Both WT and RAG1^−/−^ mice colonized with str. 630 and then challenged with the highly virulent str. VPI 10463 are protected from weight loss, whereas mice that had no exposure to str.630 experienced significant weight loss. Str. VPI 10463-challenged and str. 630-colonized RAG1^−/−^ versus VPI-challenged naive RAG1^−/−^, *P* < 0.05; str. VPI 10463-challenged and str. 630-colonized WT versus str. VPI 10463-challenged naive WT, *P < *0.01; no statistical difference was detected in comparisons of the same treatment between the two genotypes. (B) Toxin titer from intestinal content of mice in panel A as measured by Vero cell cytotoxicity assay. Both WT and RAG1^−/−^ mice colonized with str. 630 and then challenged with str. VPI 10463 have a lower toxin titer relative to naive mice challenged with str. VPI 10463. Str. VPI 10463-challenged and str. 630-colonized RAG1^−/−^ versus str. VPI 10463-challenged naive RAG1^−/−^, *P < *0.05; str. VPI 10463-challenged and str. 630-colonized WT versus str. VPI 10463-challenged naive WT, *P < *0.001. Statistical significance was calculated using a Wilcoxon test. Limit of detection was 2.3; however, for visual clarity, samples with an undetected toxin titer were plotted below the limit of detection. (C) Histopathology scoring of the colon damage of mice from panel A. Smaller panels depict scores for each component of the summary score. Summary scores of str. VPI 10463-challenged and str. 630-colonized RAG1^−/−^ versus str. VPI 10463-challenged naive RAG1^−/−^, *P < *0.05; str. VPI 10463-challenged and str. 630-colonized WT versus str. VPI 10463-challenged naive WT, *P < *0.001. For all panels, statistical significance was calculated using a Wilcoxon test with Benjamini-Hochberg corrections when appropriate. Data are from two independently run experiments with multiple cages per each treatment group.

Since protection was correlated with both precolonization and the development of an adaptive immune response to the toxins, we next tested if adaptive immunity was the sole factor preventing disease in our model by utilizing mice defective in recombination-activating gene 1 (RAG), a gene that is critical in the development of B and T cells. RAG1^−/−^ lacks the adaptive arm of its immune system (16). Following 40 days of colonization, mice were given an intraperitoneal injection of clindamycin and then challenged with spores of the lethal str. VPI 10463. Surprisingly, both the RAG^−/−^ and wild-type (WT) mice precolonized with str. 630 were protected following the challenge, while the naive mice of both genotypes succumbed to the infection (Fig. 3A and B, naive versus colonized; *P < *0.05). Scoring of the colonic pathology demonstrated that both the RAG1^−/−^ and WT mice precolonized with str. 630 had less pathology relative to the naive mice (Fig. 3C, naive versus colonized; *P < *0.05). These data demonstrate that in the absence of adaptive immunity, precolonization with C. difficile is sufficient to protect from lethal infection with spores of another strain. Furthermore, protection from severe disease in mice precolonized with str. 630 is not mediated by adaptive immunity.

### Protection afforded by low-virulence C. difficile strain develops rapidly and depends on limiting colonization of the lethal strain.

**FIG 4:**
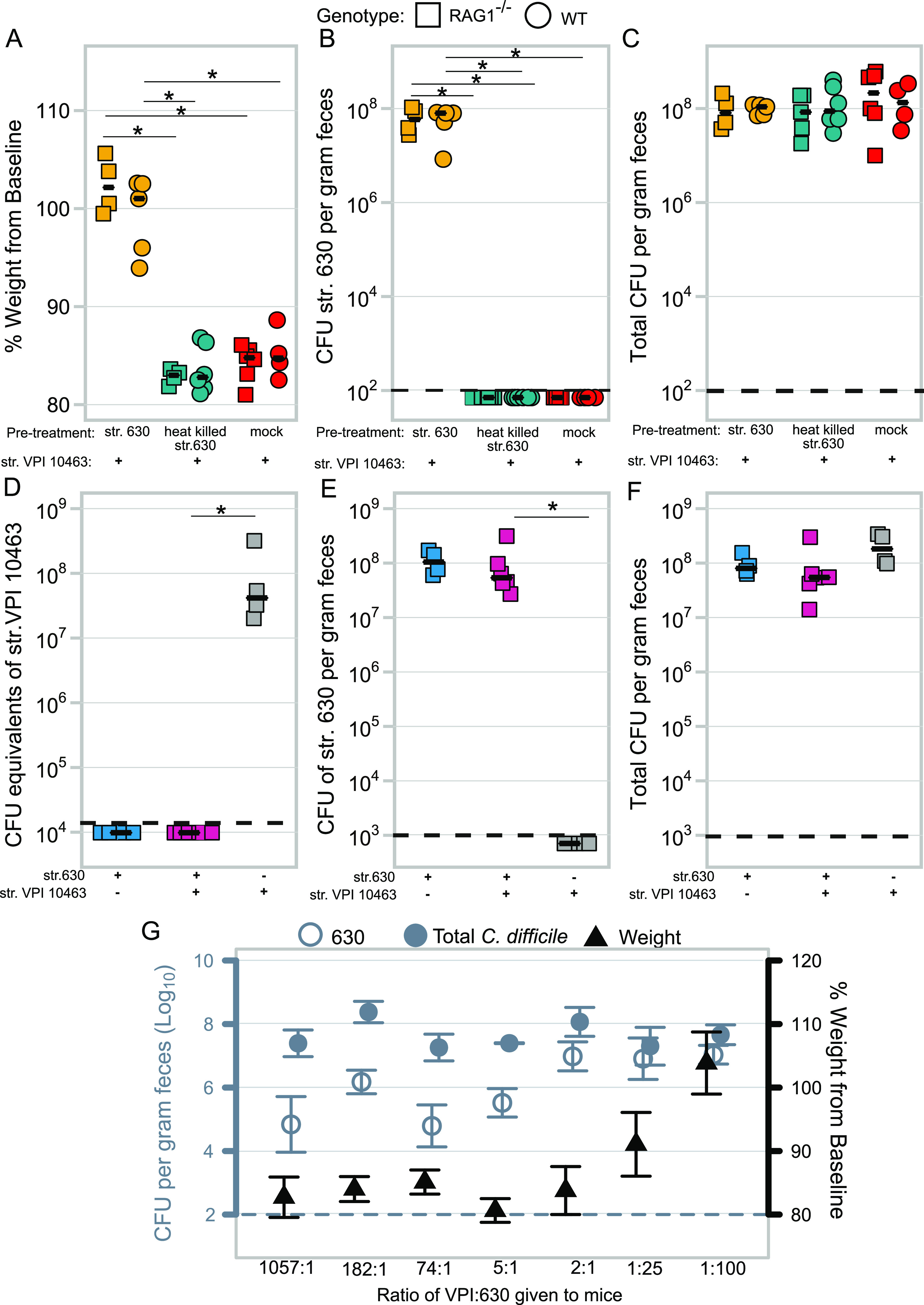
C. difficile str. 630 protects by limiting colonization of the lethal strain. (A) Change in weight at time of necropsy (day 3) relative to weight on day of challenge in mice pretreated with viable str.630, heat-killed str. 630, or water (mock). All mice were infected with str. VPI 10463 1 day following pretreatment. Both RAG1^−/−^ or WT mice given viable str. 630 did not lose weight following challenge with str. VPI 10463 compared to mice who received heat-killed str. 630 or mock; *P < *0.05 for all comparisons shown. (B) Levels of str. 630 in mice at conclusion of experiment on day 3 (str. 630 is erythromycin resistant, while str. VPI 10463 is sensitive to the antibiotic). Colonization by str. 630 was significantly different in mice given viable str. 630 from mice given heat-killed str. 630 or mock. RAG1^−/−^ mice given str. 630 versus heat-killed str. 630, *P < *0.05, or versus mock, *P < *0.05. WT mice given str. 630 versus heat-killed str. 630, *P < *0.05, or versus mock, *P < *0.05. There was no significant difference between colonization in the mock versus heat-killed str. 630 treatments for either genotype. LOD is 100 CFU/g feces; undetected samples were plotted below the LOD for visual clarity. (C) Total levels of C. difficile colonization at conclusion of experiment. There was no significant difference in total C. difficile colonization between any of the groups; *P > *0.7. (D) CFU equivalents of str. VPI 10463 in gnotobiotic mice as determined by qPCR on day 3. Mice precolonized with C. difficile str. 630 have undetectable levels of str. VPI 10463 using this assay. LOD is 1.39 × 10^4^ CFU; levels of str. VPI 10463 in str. 630-precolonized mice versus str. VPI 10463-only mice, *P < *0.01. (E) CFU/g of feces of C. difficile strain str. 630 in gnotobiotic across groups as determined by selective quantitative culture. Mice only challenged with str. VPI 10463 were not colonized with str. 630. LOD is 1,000 CFU; *P < *0.05. (F) Total CFU/g of feces of C. difficile in gnotobiotic as determined by quantitative culture on day 3. (G) Mice challenged simultaneously with both strains can be colonized by both strains. Left axis represents log_10_ CFU of total C. difficile (closed circle) or str. 630 2 days postchallenge (open circle). Right axis depicts percentage of baseline weight 2 days postchallenge (triangle). Each different inoculum ratio was given to one cage of five mice. Points represent the median value for each treatment, while the bars represent the upper and lower quartiles. For all panels in the figure, squares represent RAG1^−/−^ mice, while circles represent wild-type (WT) mice. For the data included in each figure, statistical significance was calculated using a Wilcoxon test and corrected with a Benjamini-Hochberg correction. The dashed line represents the limit of detection for each assay; for visual clarity, samples that were below the limit of detection were plotted below the line.

Having excluded the contribution of the adaptive immune response in our model, we next tested if protection required treatment with live C. difficile. Activation of innate immune pathways with microbe-associated molecular patterns, such as Toll-like receptor 5 with flagellin, protects against acute CDI (17, 18). Additionally, in other colitis models, both viable and heat-killed probiotic strains ameliorate disease via stimulation of host innate immune pathways (19, 20). Thus, we tested if defense against severe CDI could be conferred by pretreatment of mice with a high dose of heat-killed vegetative str. 630. As we had not observed a role for adaptive immunity, we shortened the model from 42 days of preinfection with str. 630 to 1 day. However, since we were additionally testing heat-killed versus live colonization, we included RAG1^−/−^ mice in this experiment to confirm findings from our persistent colonization model. Cefoperazone-treated mice were challenged with str. 630 spores, given the equivalent of 10^9^ CFU of autoclaved str. 630, or mock infected. Twenty-four hours later, all mice were challenged with str. VPI 10463 spores (Fig. S2A). We observed no protective effect of heat-killed str. 630, indicating that protection requires colonization with C. difficile str. 630, not merely exposure to antigen (Fig. 4A; *P < *0.05 for all comparisons where significance is indicated). In this short infection model, we repeated our finding that adaptive immunity is not necessary for protection, as both RAG1^−/−^ and WT mice colonized with str. 630 were protected from weight loss after just 24 h of colonization. Mice given viable C. difficile str. 630 were highly colonized with the strain, while str. 630 was not detected in mice that received the heat-killed str. 630 or mock infection (Fig. 4B). Total levels of C. difficile were not significantly different between the groups (Fig. 4C; *P > *0.7). These data suggested that precolonization with str. 630 protects by limiting colonization of str. VPI 10463.

10.1128/mBio.00522-21.2 FIG S2 Short-term infection timelines. (A) Schematic of the short-term colonization in SPF mice related to data in main text (Fig. 4A to C). (B) Schematic for experiments in germfree mice related to main text (Fig. 4D to F). (C) Schematic for experiments performing simultaneous challenge with different ratios of strains 630 and VPI 10463 spores related to main text (Fig. 4G and Fig. S4). Download FIG S2, EPS file, 1.6 MB. Copyright © 2021 Leslie et al. 2021 Leslie et al. https://creativecommons.org/licenses/by/4.0/ This content is distributed under the terms of the Creative Commons Attribution 4.0 International license.

Since treatment with viable C. difficile was required for protection, we sought to establish if this was mediated directly by str. 630 rather than indirectly through changes in the microbiota. To test this, we utilized gnotobiotic mice. Using our selective plating scheme, it is only possible to differentiate str. 630 from total C. difficile, as we were unable to identify an antibiotic that only str. VPI 10463 was resistant to. To overcome this limitation, we developed a quantitative PCR assay using primers that amplify a target in str. VPI 10463 that is absent in str. 630 (Fig. S3A and B). While these primers are not specific to solely str. VPI 10463 when used in the context of the rest of the gut microbiota, they could be used in gnotobiotic mice. RAG1^−/−^ germfree mice were either infected with str. 630 spores or left germfree; the following day, the germfree mice, in addition to one of the groups of str. 630-monoassociated mice, were challenged with str. VPI 10463 spores (Fig. S2B). Using this model, we were able to determine that precolonization with str. 630 is sufficient to prevent colonization by spores of the lethal strain, as we were unable to detect str. VPI 10463 genomic DNA in the mice that were cochallenged despite high overall levels of C. difficile measured by quantitative culture (Fig. 4D to F).

10.1128/mBio.00522-21.3 FIG S3 Validation of primers for str. VPI 10463. (A) Plot of *C_q_* (or crossing threshold) versus dilution of genomic DNA from str. VPI 10463; *R*^2^ = 0.9929. (B) The resulting PCR products formed one melt peak. Download FIG S3, EPS file, 1.6 MB. Copyright © 2021 Leslie et al. 2021 Leslie et al. https://creativecommons.org/licenses/by/4.0/ This content is distributed under the terms of the Creative Commons Attribution 4.0 International license.

Reports of patients infected with multiple strains of C. difficile suggest that despite our results, infection with multiple strains can occur (21, 22). When we infected mice with different ratios of spores from each of the two strains simultaneously, we found that both strains were capable of colonizing despite starting with over 1,000 fewer spores of str. 630 (Fig. S2C). While both strains were detectable when coinoculated, protection, defined as a lack of weight loss and low clinical scores, was not observed unless str. 630 was the dominant strain (Fig. 4G; Fig. S4Ato D). Interestingly, when infecting with different ratios of the two strains, the total burden would not pass a threshold of 10^9^, suggesting a population-carrying capacity for C. difficile in the mouse gut. Together, these data demonstrate that protection from lethal disease requires colonization with high levels of str. 630 to prevent establishment of the second strain.

10.1128/mBio.00522-21.4 FIG S4 Plots from coinoculation experiments show that high levels of strain 630 correlate with protection from disease. (A) Spearman correlation analysis of percentage of baseline weight versus log_10_ CFU of C. difficile str. 630 at 2 days postchallenge. (B) Spearman correlation analysis of total clinical score versus log_10_ CFU of C. difficile str. 630 at 2 days postchallenge. (C) Spearman correlation analysis of % of baseline weight versus log_10_ total C. difficile CFU on day 2 postchallenge. (D) Spearman correlation analysis of total clinical score versus log_10_ total C. difficile CFU on day 2 postchallenge. Download FIG S4, EPS file, 2.0 MB. Copyright © 2021 Leslie et al. 2021 Leslie et al. https://creativecommons.org/licenses/by/4.0/ This content is distributed under the terms of the Creative Commons Attribution 4.0 International license.

### Limitation of the lethal strain is mediated by decreased availability of a cogerminant, glycine.

**FIG 5:**
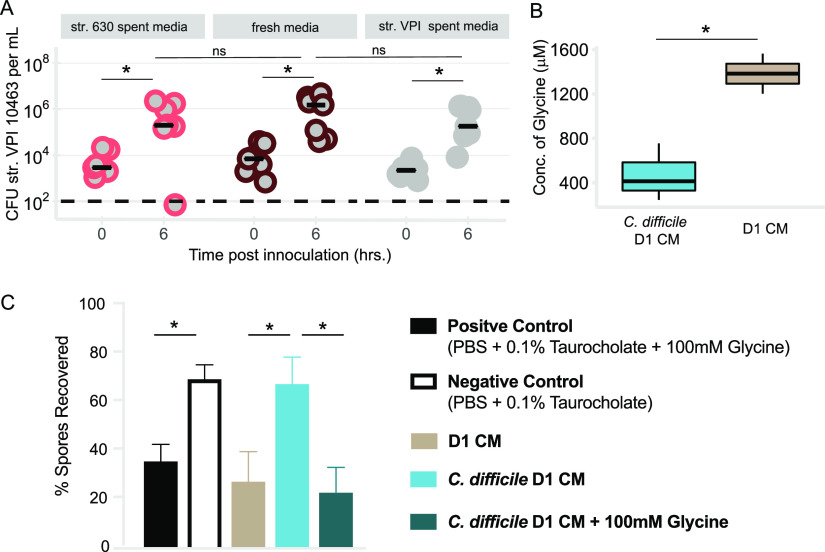
Colonization with C. difficile str. 630 significantly reduces the cogerminant glycine in cecal contents, leading to reduced germination of invading strain. (A) Spent cecal medium from 24 h growth of str. 630 *ex vivo* supports robust growth of str. VPI 10463. Filter-sterilized spent cecal medium was inoculated with vegetative str. VPI 10463, and colonization was monitored by quantitative culture at time zero and 6 h. Cecal medium that grew str. 630 supported robust growth of str. VPI 10463. Str. VPI 10463 colonization at time (*t*) of 0 versus *t* of 6 h in str. 630 spent culture, *P < *0.05; str. VPI 10463 colonization at *t* of 0 versus *t* of 6 h in fresh medium, *P < *0.01; str. VPI 10463 colonization at *t* of 0 versus *t* of 6 h in str. VPI 10463 spent culture media, *P < *0.01. Data represent two independent experiments using separate batches of cecal medium (pooled from at least 6 mice for each batch) run in at least triplicate. Black bars represent median. (B) Concentration of glycine (μM) in cecal medium made from cecal contents of mice 1 day postinfection with C. difficile (C. difficile D1 CM) compared to cecal medium made from mock-challenged mice (D1 CM), *P < *0.05. Data represent values from two separate batches of cecal medium per group. (C) *Ex vivo* germination of str. VPI 10463 spores in cecal medium from panel B. PBS supplemented with 0.1% sodium taurocholate served as a negative control, while PBS supplemented with 0.1% sodium taurocholate and 100 mM glycine served as a positive control. Bars represent mean with SEM. Negative control versus positive control, *P < *0.05; D1 CM versus C. difficile D1 CM, *P < *0.05; C. difficile D1 CM versus C. difficile D1 CM plus 100 mM glycine, *P < *0.05. Data are from at least two independent experiments run in at least duplicate. Statistical significance was calculated using a Wilcoxon test with Benjamini-Hochberg correction (panel A), *t* test (panel B), or analysis of variance (ANOVA) with Sidak’s multiple-comparison test (panel C).

Others have reported that precolonization with one strain of C. difficile provides protection from challenge with a more virulent strain (23–26). The prevailing hypothesis is that consumption of nutrients by the first strain limits the ability of the invading strain to grow (12). We tested this hypothesis in an *ex vivo* assay using sterile medium prepared from the cecal contents of susceptible mice. Using this approach, we found that when vegetative C. difficile was inoculated into susceptible mouse cecal medium, both strains displayed significant growth after 24 h (Fig. S5A; *P* < 0.001). To test if 1 day of colonization was sufficient to reduce the nutrients required for growth, we added vegetative str. VPI 10463 to filter-sterilized spent culture from the experiments in Fig. S3A. Spent cecal medium from 24-h cultures of both str. 630 and str. VPI 10463 supported another round of significant growth (Fig. 5A). To test if nutrient utilization by str. 630 was different *in vivo*, we additionally assessed growth of str. VPI 10463 in cecal medium made from mice infected with str. 630 for 24 h. This medium also supported robust growth, demonstrating that in both batch culture and *in vivo*, 24 h of colonization is not sufficient to reduce the nutrients required for growth of a vegetative invading strain (Fig. S5B). Additionally, as we were able to culture vegetative str. VPI 10463 in the spent culture of str. 630, we excluded the possibility of inhibition due to secreted products like bacteriocins or phage in this model. This was confirmed by agar overlay assays (data not shown).

10.1128/mBio.00522-21.5 FIG S5 *Ex vivo* growth in cecal medium. (A) Sterile cecal filtrate from susceptible mice (day 0) was inoculated with vegetative cells of C. difficile str. 630, vehicle, or vegetative cells of C. difficile str. VPI 10463. Levels of colonization were monitored by quantitative culture at time 0 and 24 h. Cecal medium supports robust growth of both strains; *P < *0.001. Data are from two independent experiments run in at least triplicate using separate batches of cecal medium. (B) Growth of C. difficile str. VPI 10463 in cecal medium made from content from mice infected for 24 h with C. difficile str. 630 (630 CM). Strain VPI 10463 colonization at *t* of 0 versus *t* of 24 h; *P < *0.05. Statistical significance for all comparisons was calculated using a Wilcoxon test. Download FIG S5, EPS file, 1.7 MB. Copyright © 2021 Leslie et al. 2021 Leslie et al. https://creativecommons.org/licenses/by/4.0/ This content is distributed under the terms of the Creative Commons Attribution 4.0 International license.

Our group previously observed that 18 h of colonization with strain 630 had significantly decreased levels of amino acids relative to susceptible mice (27). Vegetative C. difficile can utilize certain amino acids to enhance growth (28–30). Some of the same amino acids used for growth can also serve as spore cogerminant (31). Since the primary infectious form of C. difficile is not the vegetative cell but rather the environmentally stable spore, we hypothesized that decreased amino acids might mediate diminished levels of germination and thus limit colonization by the invading strain (32). Using targeted metabolomics, we measured concentrations of amino acids in cecal medium (CM) made from mice that had been off cefoperazone for 3 days (D1 CM) compared to mice that had been off cefoperazone for 2 days followed by infection with C. difficile for 24 h (C. difficile D1 CM). Twenty-four hours of colonization with str. 630 (or a nontoxigenic strain) results in a significant reduction of glycine relative to mock-challenged-susceptible animals (Fig. 5B; Fig. S6). Glycine is one of the most effective amino acid signals for germination of C. difficile spores (33). As we had already ruled out limitation of vegetative growth, we asked if this decrease in glycine altered germination of str. VPI 10463 spores. Germination was assessed by incubation of spores in a given condition for 15 min followed by heat treatment to kill any cells that germinated. If germination occurred, then the postheat CFU would be lower than the preheat amount; if there was minimal germination, the spores would survive heating, and levels would remain constant between the pre- and post-time points.

10.1128/mBio.00522-21.6 FIG S6 Targeted metabolomics measuring amino acids in cecal medium from mock-challenged or C. difficile-colonized mice. Concentration (μM) of amino acids in cecal medium (CM). D1 CM is medium made from cecal contents of mice that were made susceptible with antibiotics and then mock challenged. C. difficile D1 CM is medium from cecal contents of mice that were colonized with a low-virulence strain of C. difficile for 24 h. Data represent at least two different batches of cecal medium per condition; each batch was made from at least 6 pooled cecal contents. Statistical significance for all comparisons was calculated using a *t* test. Download FIG S6, EPS file, 0.8 MB. Copyright © 2021 Leslie et al. 2021 Leslie et al. https://creativecommons.org/licenses/by/4.0/ This content is distributed under the terms of the Creative Commons Attribution 4.0 International license.

Heating the spores incubated in phosphate-buffered saline (PBS) supplemented with 0.1% taurocholate and 100 mM glycine resulted in a significant reduction in CFU, with only 35% of spores recovered, indicating robust germination (Fig. 5C). However, heating spores incubated in PBS plus taurocholate resulted in minimal changes between the pre- and postlevels, with 70% of spores recovered, suggesting minimal germination. C. difficile str. VPI 10463 spores incubated in D1 CM resulted in recovery of 27% of spores, suggesting that this medium supports germination. However, when spores were incubated in C. difficile D1 CM, 67% of spores were recovered, suggesting that this medium does not support robust germination. C. difficile D1 CM enabled significantly less germination than D1 CM (*P < *0.05). To determine if the observed decrease in germination of str. VPI 10463 spores in C. difficile D1 CM was due to lower glycine, we tested if addition of exogenous glycine could rescue germination. Addition of 100 mM glycine to C. difficile D1 CM significantly decreased the percentage of spores recovered compared to C. difficile D1 CM alone (*P < *0.05).

To provide *in vivo* support for the hypothesis that the protection against infection with VPI 10463 afforded by precolonization with str. 630 was due to decreased germination of the more virulent strain, we tested if challenging mice with vegetative str. VPI 10463 overcame this colonization resistance. Specific-pathogen-free (SPF) mice were made susceptible to infection and then challenged with str. 630 spores; the following day, the mice were challenged with either 6 × 10^4^ CFU of str. VPI spores or 1 × 10^7^ CFU of str. VPI 10463 vegetative cells (Fig. S7A). We used a higher dose of vegetative cells because, in our hands, over 1 × 10^5^ CFU of vegetative cells is necessary to generate a severe infection within 2 days of challenge, the timeline we were observing with our previous experiments in this study (13). Two days after challenge with the lethal strain, mice given vegetative str. VPI 10463 cells had a modest but significantly higher clinical score than animals challenged with spores (Fig. S7B). Together, these results demonstrate that precolonization with str. 630 reduces levels of glycine in the gut, leading to a reduction in the ability of a second strain to germinate.

10.1128/mBio.00522-21.7 FIG S7 Challenge with vegetative str. VPI 10463 abrogates protection by precolonization with str. 630. (A) Schematic for experiment challenging mice with vegetative cells or spores of strain VPI 10463. Male and female mice were made susceptible with cefoperazone and then precolonized with str. 630. The following day, mice were challenged with either spores (*n *= 6, two cages) or vegetative cells (*n* = 7, two cages) of strain VPI 10463. (B) Box plots showing clinical signs of disease (a score which encompasses weight loss, movement, appearance, and diarrhea) were significantly elevated on day 3 of the experiment; *P < *0.05. Statistical significance was calculated using a Wilcoxon test. Download FIG S7, EPS file, 0.6 MB. Copyright © 2021 Leslie et al. 2021 Leslie et al. https://creativecommons.org/licenses/by/4.0/ This content is distributed under the terms of the Creative Commons Attribution 4.0 International license.

## DISCUSSION

The role of the gut microbiota in limiting colonization by C. difficile has been appreciated for over 3 decades; however, how the microbiota provides colonization resistance remains to be fully elucidated (12, 23). Many studies have focused on a top-down approach to identify and create defined consortia that confer the same protection as the intact community (34, 35). We took an alternative approach and built off the observation that administration of a single bacterium (nontoxigenic C. difficile) limited subsequent colonization by another strain of C. difficile (10, 36). We sought to determine the mechanisms by which precolonization with one strain of C. difficile protects from infection with another. We hypothesized that protection was the result of both intraspecific bacterial competition and the development of host immunity to C. difficile antigens, including the toxins. To evaluate this, we utilized two well-characterized lab strains that, despite being differentially virulent in our mouse model, express nearly identical forms of both TcdA and TcdB (37).

Using multiple infection models, we determined that precolonization with a less virulent strain is sufficient to protect from challenge with a lethal strain of C. difficile, even in the absence of adaptive immunity. Additionally, we showed that protection is dependent on high levels of colonization by the less virulent strain, and str. 630 alone is sufficient to limit colonization of the invading strain. While we observed complete exclusion of str. VPI 10463 in the gnotobiotic mice, since we were unable to directly monitor levels of str. VPI 10463 in our SPF mice, we were unable to determine if precolonization with str. 630 completely excludes str. VPI 10463 in the context of a more complex microbiota. The prevailing hypothesis has been that precolonization with one strain of C. difficile limits vegetative growth of the challenging strain. Our results question this model, as 24 h of growth by one strain is not sufficient to deplete nutrients such that it prevented vegetative growth of a second strain.

While other bacterial therapies for C. difficile infection, such as fecal microbiota transplants, can lead to clearance of C. difficile from the gut, we were unable to use str. 630 to “treat” mice already colonized with the lethal strain (Fig. S8) (6). Recently, another group reported that a different toxigenic but low-virulence strain of C. difficile could both protect and “treat” infection with str. VPI 10463 (25). This highlights an important consideration when designing bacterial-based therapies for treatment of CDI, as unique strains have different relative fitness. The ability to detect low levels of str. 630 in our coinoculation model suggests that the strains are able to segregate niches; however, more work will be needed to fully elucidate which nutrients are preferred by each strain. Additionally, experiments focused on development of microbial therapies to restore colonization resistance against C. difficile should seek to understand the metabolic pathways that enable certain strains to outcompete established C. difficile versus inhibit colonization of invading strains as has been done with Escherichia coli in the streptomycin mouse model (38, 39).

10.1128/mBio.00522-21.8 FIG S8 Treatment with strain 630 does not rescue mice previously infected with strain VPI 10463. Wild-type mice were challenged with strain VPI 10463 spores and the next day either challenged with strain 630 (*n*= 5) or mock (*n* = 4). Treatment with str. 630 did not protect against weight loss, and mice had to be euthanized. *P > *0.05; statistical significance was calculated using a Wilcoxon test. Download FIG S8, EPS file, 0.5 MB. Copyright © 2021 Leslie et al. 2021 Leslie et al. https://creativecommons.org/licenses/by/4.0/ This content is distributed under the terms of the Creative Commons Attribution 4.0 International license.

The major finding from this study is that reduction of amino acids, specifically glycine, following colonization with one strain of C. difficile is sufficient to decrease germination of the second strain. This suggests that the axis of intraspecific competition for nutrients includes multiple life stages of C. difficile. Although this type of indirect competition across multiple life stages has been well recognized in macroecology, to our knowledge, it has not yet been applied to the study of C. difficile infection (40, 41). To date, studies evaluating the role of the microbiota in altering germination of C. difficile spores *in vivo* have primarily focused on bile acids, specifically the primary bile acid taurocholate and the secondary bile acid deoxycholate (2, 34, 35). While bile acids play important roles in germination, vegetative growth, and toxin activity, this work demonstrates that microbial metabolism of other nutrients can also affect germination (42). Furthermore, these results suggest that targeting nutrients used by all life stages, including those that are metabolically inactive (such as a spore), could be an improved strategy when developing bacterial therapeutics that aim to restore colonization resistance in the gut.

## MATERIALS AND METHODS

### Animals and housing.

Both male and female mice aged 5 to 12 weeks were used in these studies. The wild-type (WT) C57BL/6 specific-pathogen-free (SPF) mice were from a breeding colony originally derived from the Jackson Laboratory nearly 20 years ago. The RAG1^−/−^ (B6.129S7-*Rag1^tm1Mom^*/J) SPF mice were from a breeding colony that started with mice from the Jackson Laboratory in 2013. Germfree RAG1^−/−^ mice were obtained from a colony established and maintained by the University of Michigan germfree facility.

Animal husbandry was performed in an AAALAC-accredited facility. Animals were housed with autoclaved cages, bedding, and water bottles. Mice were fed a standard irradiated chow (LabDiet; catalog no. 5LOD) and had access to food and water *ad libitum*. Cage changes were performed in a biological safety cabinet. To prevent cross-contamination between cages, hydrogen peroxide-based disinfectants in addition to frequent glove changes were utilized during all manipulation of SPF animals. A chlorine-based disinfectant was used during manipulation of the germfree mice. The frequency of cage changes varied depending on the experiment. All mice were maintained under a cycle of 12 h of light/dark in facilities maintained at a temperature of 72°C ± 4°. Animal sample size was not determined by a statistical method. Multiple cages of animals for each treatment were used to control for possible differences in the microbiota between cages. Mice were evaluated daily for signs of disease; those determined to be moribund were euthanized by CO_2_ asphyxiation. The University Committee on the Care and Use of Animals at the University of Michigan approved this study.

### Preparation of spore or vegetative inocula of C. difficile.

Spore stocks of C. difficile strains 630 (ATCC BAA-1382) and VPI 10463 (ATCC 43255) were prepared as previously described (15).

Vegetative C. difficile was cultured and manipulated in a vinyl anaerobic chamber (Coy Laboratory Products) at 37°C. To generate the vegetative cell inoculum, strain VPI 10463 was streaked from the spore stock onto a plate of prereduced cycloserine-cefoxitin-fructose agar containing 0.1% taurocholate (TCCFA). TCCFA was prepared as previously described (15). The following day, a colony from the TCCFA was subcultured onto brain heart infusion (BHI) agar. After overnight growth, a colony from this plate was inoculated into 5 ml of brain heart infusion broth supplemented with 0.01% cysteine (BHIS) and incubated overnight. The following morning, broth-grown bacteria were harvested by centrifugation at 4,000 × *g* for 13 min. The supernatant was discarded, and the pellet was resuspended in 5 ml of sterile phosphate-buffered saline (PBS) (Gibco; catalog no. 10010023). The pellet was washed two more times before being used to challenge mice.

### Preparation of autoclaved C. difficile.

Heat-killed C. difficile strain 630 was made from an overnight culture grown at 37°C in BHIS broth. The culture was enumerated by plating for CFU per ml^−1^. Broth-grown bacteria were harvested by centrifugation, and the cell pellet was washed and resuspended in PBS at a density of 2.7 × 10^10^ CFU per ml^−1^. The suspension was autoclaved at 121°C and 14 lb/in^2^ for 30 min to kill the vegetative cells and inactivate any spores. In experiments using heat-killed C. difficile, mice received 10^9^ CFU equivalents in 0.05 ml. A portion of the sample was cultured on prereduced TCCFA to confirm that the inactivation was successful.

### Infections.

In experiments using both WT and RAG1^−/−^ SPF mice, age- and sex-matched mice were cohoused starting at 3 weeks of age for 33 days through antibiotic administration. Upon infection, animals were separated into single genotype housing.

All SPF mice received the antibiotic cefoperazone (MP Pharmaceuticals; catalog no. 0219969501) dissolved in Gibco distilled water at a concentration of 0.5 mg/ml administered *ad libitum* for 10 days (15). While mice were on antibiotics, the water was changed every 2 days. Following completion of antibiotics, mice were given plain Gibco distilled water for 2 days before challenge with either spores or water (mock). C. difficile spores suspended in 50 to 100 μl of Gibco distilled water were administered via oral gavage. The number of viable spores in each inoculum was enumerated by plating for CFU per ml^−1^ on prereduced TCCFA. Mice were infected with between 10^3^ and 10^4^ spores of strain 630. Over the course of the infection, mice were weighed routinely, and stool was collected for quantitative culture.

In our long-term colonization model, 41 days after primary infection, mice were given an intraperitoneal injection (i.p.) of clindamycin (Sigma; catalog no. C5269) in sterile saline at a concentration of 10 mg/kg to perturb the gut microbial community as described previously (6, 13). The next day, mice were either mock challenged with water or with between 10^4^ and 10^5^ spores from str. VPI 10463. Mice were euthanized 2 days postinfection with str. VPI 10463 (day 44 of the model), and samples for quantification of colonization, toxin cytotoxicity, and histopathology were collected.

In the short-term infection model, mice were challenged with spores, heat-killed str. 630, or vehicle on day 0 and, on the following day, challenged with spores or vegetative cells of strain VPI 10463 (see Fig. S2 and S7A) for experiment-specific timelines.

In the simultaneous coinfections, mice were challenged with different ratios of str. 630 and str. VPI 10463 within a total amount of 10^4^ spores. During infections, when indicated, mice were scored for clinical signs of disease based on criteria previously described by reference 43.

At the conclusion of each experiment, mice used in dual-genotype experiments were genotyped using DNA from an ear snip using primers and cycling conditions as outlined by the Jackson Laboratory.

### Quantitative culture from intestinal content.

Fecal pellets or colonic content were collected from each mouse into preweighted sterile tubes. Following collection, the tubes were reweighed and passed into an anaerobic chamber (Coy Laboratories). In the chamber, each sample was diluted 1 to 10 (wt/vol) using prereduced sterile PBS and serially diluted. We spread 100 μl of a given dilution onto prereduced TCCFA or, when appropriate, TCCFA supplemented with either 2 or 6 μg/ml of erythromycin (Sigma; catalog no. E0774). Strain 630 is erythromycin resistant; use of 2 μg/ml of erythromycin in TCCFA plates reduced background growth from other bacteria in the sample, while TCCFA with 6 μg/ml of erythromycin enabled selection of str. 630 (erythromycin resistant) from str. VPI 10463 (erythromycin sensitive). Plates were incubated at 37°C in the anaerobic chamber, and colonies were enumerated at 18 to 24 h. Since taurocholate was used in these plates, the colony counts represent both vegetative cells and spores in each sample. Plates that were used to determine if mice were negative for C. difficile were held for 48 h.

### Toxin cytotoxicity assay.

Intestinal content was collected from each mouse into a preweighted sterile tube and stored at −80°C. At the start of the assay, each sample was diluted 1:10 weight per volume using sterile PBS. Following dilution, the sample was filter sterilized through a 0.22-μm filter, and the activity of the toxins was assessed using a Vero cell rounding-based cytotoxicity assay as described previously (6, 44). The cytotoxicity titer was determined for each sample as the last dilution, which resulted in at least 80% cell rounding. Toxin titers are reported as the log_10_ of the reciprocal of the cytotoxicity titer.

### Histopathology evaluation.

Mouse ceca and colon tissue were saved in histopathology cassettes and fixed in 10% formalin, followed by storage in 70% ethanol. McClinchey Histology Labs, Inc. (Stockbridge, MI) prepared the tissue, including embedding samples in paraffin, sectioning, and generation of hematoxylin and eosin-stained slides. A board-certified veterinary pathologist scored the slides and was blinded to the experimental groups, using previously described criteria (13, 44).

### Anti-TcdA IgG ELISA.

Blood was collected from mice via saphenous vein puncture into capillary blood collection tubes. Samples were spun down, and serum was stored at −80°C. Levels of IgG specific to C. difficile TcdA were measured in serum from mice using a previously described enzyme-linked immunosorbent assay (ELISA) protocol (15) with a few modifications. Briefly, 96-well enzyme immunoassay (EIA) plates were coated with 100 μl of purified C. difficile TcdA (List Laboratories; catalog no. 152C) at 1 μg/ml in 0.05 M sodium bicarbonate buffer, pH 9.6, overnight at 4°C. Mouse serum was diluted to 1:400 in blocking buffer and serially diluted 1:3. Each sample was run in duplicate. Negative controls included preimmune sera from the mice as well as serum-negative wells. A positive control consisting of a monoclonal mouse anti-TcdA IgG antibody was run on each plate. The optical density at 410 nm (OD_410_) was recorded on a VersaMax plate reader (Molecular Devices, Sunnyvale CA). The anti-toxin A (TcdA) IgG titer was defined as the last dilution where both replicates had an OD_410_ greater than mean absorbance of all negative wells on the plate plus three times the standard deviation from that mean.

### Serum neutralizing anti-toxin antibody assay.

The serum-neutralizing anti-toxin antibody assay was based on a previously described assay (7) with the following modifications: the concentration of purified TcdA or TcdB (List Laboratories) that resulted in 100% cell rounding was determined empirically, using the toxin activity assay. Vero cells were seeded at a density of 10^5^ cells/0.075 ml per well onto tissue culture-treated plates. Four times the concentration of toxin that resulted in 100% cell rounding (64 μg/ml for TcdA and 4 μg/ml for TcdB) was incubated with serial dilutions of serum from mice. The serum toxin mixture was added to Vero cells and incubated overnight. The neutralizing titer was determined to be the last dilution which had less than 50% of round cells in the well. Each sample was run in duplicate, and the results from discordant wells were averaged. Toxin-only wells served as negative controls, while goat anti-TcdA and anti-TcdB serum served as a positive control (TechLab; catalog no. T5015). Sera from mice before they were infected were run as an additional negative control.

### Quantitative PCR.

Primer3 (45) was used to design primers that differentiated strain VPI 10463 (IMG Genome ID 2512047057) from strain 630 (GenBank accession no. AM180355.1) using publicly available genomes. While the primers differentiated between the strains in pure culture, they also picked up other bacteria in the context of the microbiota and were only used in gnotobiotic experiments. C. difficile str. VPI 10463 primers used were forward, 5′-TTTCACATGAGCGGACAGGC-3′, and reverse, 5′-TCCGAAGGAGGTTTCCGGTT-3′. The expected product size is 153 nucleotides, and the optimal annealing temperature was empirically determined to be 56°C.

For quantitative PCR, DNA from fecal samples was diluted in ultrapure water such that 20 ng of DNA was added to each reaction. Each sample was run in triplicate. Samples were loaded into a LightCycler 480 multiwell plate (Roche; product no. 04729692001), with FastStart essential DNA green master mix (Roche; product no. 06402712001) and 0.5 μM each primer. The plate was sealed with optically clear sealing tape, briefly spun down, and run on the Roche LightCycler 96. The following conditions were used for quantitative PCR (qPCR): 95°C for 10 min, followed by 30 cycles of 95°C for 10 s, 56°C for 10 s, and 72°C for 10 s. A melt cycle was run at the conclusion of the amplification.

Genomic DNA from str. VPI 10463 from a culture with a known quantity of CFU was used to generate a standard curve. Negative controls included no-template control wells in addition to dilutions of genomic DNA from str. 630.

### Preparation of cecal medium.

Cecal medium was prepared from SPF mice that were treated for 10 days with cefoperazone. Uninfected mice were sacrificed two (day 0 postchallenge) or 3 days (equivalent to day 1 postchallenge) after the cessation of the antibiotics. Cecal medium was also made from infected mice, following our antibiotic regimen, 2 days after the completion of antibiotics mice were infected with C. difficile and sacrificed 1 day later (C. difficile day 1). Following euthanasia, the cecum was removed, and the content was harvested into a sterile 50-ml conical tube. The tube was spun at 3,000 rpm at room temperature (RT) for 10 min to separate the solid matter from the liquid portion. The liquid portion of the sample was removed and diluted 1:2 by volume in sterile PBS without calcium or magnesium (Gibco). The sample was spun again at 3,000 rpm for 5 min at RT; the liquid portion was then sterilized using filters with successively smaller pore sizes (from 0.8 μm to 0.45 μm and finally a 0.22-μm filter). Following passage through the 0.22-μm filter, the medium was frozen at −80°C until use. The medium was tested for sterility by inoculating into prereduced BHI in the anaerobic chamber; samples that gave rise to turbid growth after 48 h were discarded.

### *Ex vivo* vegetative growth.

To determine if 24 h of str. 630 growth depletes the gut of the nutrients required for vegetative growth of str. VPI 10463, we used an *ex vivo* approach, utilizing sterile cecal medium, prepared as described above, from susceptible mice. We thawed 180-μl aliquots of day 0 cecal medium and allowed them to equilibrate in the anaerobic chamber overnight. The inocula were prepared from an overnight culture of C. difficile (strains 630 and VPI 10463) grown in BHI plus 0.01% cysteine. Each culture was back diluted 1:10 and incubated at 37°C for 2 h. After 2 h had elapsed, 500 μl of the culture was spun down for 4 min in the anaerobic chamber using a mini-centrifuge. To prevent the introduction of nutrients from carryover BHI, the supernatant was removed, and the tubes were spun for an additional minute. Any remaining BHI was removed, and the pellets were then suspended in 500 μl of sterile anaerobic PBS. Both strains were then diluted 1:100 into sterile anaerobic PBS. Twenty microliters of this 1:100 dilution were inoculated into the cecal medium. Vegetative CFU were enumerated at time (*t*) of 0 and 24 h by plating on cycloserine-cefoxitin-fructose agar (CCFA, which lacks the germinant taurocholate). Additionally, samples were plated on BHI plus 0.01% cysteine to check for any contamination. After 24 h, the samples were removed from the chamber; cells were pelleted by centrifugation at 2,000 × g for 5 min. The supernatant was sterilized by passage through a 0.22-μm 96-well filter plate, and the sterile flowthrough was passed back into the chamber to equilibrate overnight. The following day, the VPI 10463 inoculum was prepared as described earlier and inoculated into the spent cecal medium. CFU were monitored by plating samples at *t* of 0 and 6 h on CCFA and BHI plus 0.01% cysteine.

### *Ex vivo* germination assay.

To determine if 24 h of strain 630 growth alters the ability of VPI 10463 spores to germinate, we performed an *ex vivo* germination assay based on a previously described method (46) with the following modifications. Rather than intact content, we used sterile cecal medium from mice that were off antibiotics for 3 days (D1 cecal medium) or infected with strain 630 for 24 h (C. difficile D1 cecal medium). We thawed 180-μl aliquots of the cecal medium and allowed them to equilibrate in the anaerobic chamber overnight. The next day, 10 μl of spores from strain VPI 10463 were inoculated into the medium. Controls included sterile PBS (Gibco) plus 0.1% sodium taurocholate (Sigma; catalog no. T4009) and PBS with 0.1% sodium taurocholate and 100 mM glycine (Sigma; catalog no. 50046). Following inoculation, samples were incubated anaerobically at RT for 15 min, after which approximately half the volume was immediately plated on BHI plus 0.1% sodium taurocholate plus 0.01% cysteine agar plates, and the tubes were passed out of the chamber and placed in a water-filled heat block set at 65°C for 20 min. The heating step kills off any vegetative cells. Additional controls included plating the spore inoculum on BHI without taurocholate to check for presence of any vegetative cells in the stock as well as heating suspensions of vegetative cells to confirm efficacy of heat killing. Following the 20-minute incubation, tubes were passed back into the chamber, and the remaining sample was plated on BHI with 0.1% sodium taurocholate plus 0.01% cysteine agar plates. The CFU recorded from the preheat plate represents the entire inoculum, including remaining spores and any cells that germinated, while the postheat plate represents only remaining spores. The percentage of spores recovered was calculated as the postheat counts divided by preheat count multiplied by 100.

### Metabolomics.

Quantification of amino acid concentrations in the cecal medium by targeted metabolomics was performed by the University of Michigan Medical School Metabolomics Core. Amino acids were measured using the Phenomenex EZfaast kit. Samples were extracted, semipurified, derivatized, and measured by electron ionization-gas chromatography-mass spectrometry (EI-GC-MS) using norvaline as an internal standard for normalization as described previously (47).

### Microbial community analysis.

Genomic DNA was extracted from approximately 200 to 300 μl of fecal sample using the MoBio PowerSoil HTP 96 DNA isolation kit (formerly MoBio, now Qiagen) on the Eppendorf epMotion 5075 automated pipetting system according to the manufacturers’ instructions.

The University of Michigan Microbial Systems Laboratory constructed amplicon libraries from extracted DNA as described previously (6). Briefly, the V4 region of the 16S rRNA gene was amplified using barcoded dual-index primers as described by Kozich et al. (48). The PCR included the following: 5 μl of 4 μM stock combined primer set, 0.15 μl of Accuprime high-fidelity *Taq* with 2 μl of 10× Accuprime PCR II buffer (Life Technologies; catalog no. 12346094), 11.85 μl of PCR-grade water, and 1 μl of template. The PCR cycling conditions were as follows: 95°C for 2 min, 30 cycles of 95°C for 20 s, 55°C for 15 s, and 72°C for 5 min, and 10 min at 72°C. Following construction, libraries were normalized and pooled using the SequelPrep normalization kit (Life Technologies; catalog no. A10510-01). The concentration of the pooled libraries was determined using the Kapa Biosystems library quantification kit (Kapa Biosystems; catalog no. KK4854), while amplicon size was determined using the Agilent Bioanalyzer high-sensitivity DNA analysis kit (part no. 5067-4626). Amplicon libraries were sequenced on the Illumina MiSeq platform using the MiSeq Reagent 222 kit v2 (catalog no. MS-102-2003) (500 total cycles) with modifications for the primer set. Illumina’s protocol for library preparation was used for 2-nM libraries, with a final loading concentration of 4 pM spiked with 10% PhiX for diversity. The raw paired-end reads of the sequences for all samples used in this study can be accessed in the Sequence Read Archive under accession no. PRJNA388335. Raw sequences were curated using the mothur v1.39.0 software package (49) following the Illumina MiSeq standard operating procedure. Briefly, paired-end reads were assembled into contigs and aligned to the V4 region using the silva 16S rRNA sequence database (release v128) (50); any sequences that failed to align were removed, and sequences that were flagged as possible chimeras by UCHIME were also removed (51). Sequences were classified with a naive Bayesian classifier (52) using the ribosomal database project (RDP) and clustered into operational taxonomic units (OTUs) using a 97% similarity cutoff with the OptiClust clustering algorithm (53). The number of sequences in each sample was then rarefied to 9,000 sequences to minimize bias due to uneven sampling. Following curation in mothur, further data analysis and figure generation were carried out in R (v3.6.3) using standard and loadable packages (54).

### Statistical analysis and generation of figures.

The data and code for the analysis associated with this study are available at https://github.com/jlleslie/Intraspecific_Competition. For the purpose of distinguishing between values that were detected at the limit of detection (LOD) versus those that were undetected, all results that were not detected by a given assay were plotted at an arbitrary point below the LOD. However, for statistical analysis, the value of $\text{LOD/}\sqrt{2}$ was imputed for undetected values. Figures and statistical analysis were generated in R (v3.6.3) using standard and loadable packages or GraphPad (Prism version 8). Adobe Illustrator was used to arrange panels and generate final figures.

## References

[1] Lessa FC, Mu Y, Bamberg WM, Beldavs ZG, Dumyati GK, Dunn JR, Farley MM, Holzbauer SM, Meek JI, Phipps EC, Wilson LE, Winston LG, Cohen JA, Limbago BM, Fridkin SK, Gerding DN, McDonald LC. 2015. Burden of *Clostridium difficile* infection in the United States. N Engl J Med 372:825–834. doi:10.1056/NEJMoa1408913. 25714160 PMC10966662

[2] Theriot CM, Koenigsknecht MJ, Carlson PE, Jr., Hatton GE, Nelson AM, Li B, Huffnagle GB, Z Li J, Young VB. 2014. Antibiotic-induced shifts in the mouse gut microbiome and metabolome increase susceptibility to *Clostridium difficile* infection. Nat Commun 5:3114. doi:10.1038/ncomms4114. 24445449 PMC3950275

[3] Koenigsknecht MJ, Theriot CM, Bergin IL, Schumacher CA, Schloss PD, Young VB. 2015. Dynamics and establishment of *Clostridium difficile* infection in the murine gastrointestinal tract. Infect Immun 83:934–941. doi:10.1128/IAI.02768-14. 25534943 PMC4333439

[4] Carter GP, Rood JI, Lyras D. 2012. The role of toxin A and toxin B in the virulence of *Clostridium difficile*. Trends Microbiol 20:21–29. doi:10.1016/j.tim.2011.11.003. 22154163

[5] McDonald LC, Gerding DN, Johnson S, Bakken JS, Carroll KC, Coffin SE, Dubberke ER, Garey KW, Gould CV, Kelly C, Loo V, Shaklee Sammons J, Sandora TJ, Wilcox MH. 2018. Clinical practice guidelines for *Clostridium difficile* infection in adults and children: 2017 update by the Infectious Diseases Society of America (IDSA) and Society for Healthcare Epidemiology of America (SHEA). Clin Infect Dis 66:e1–e48. doi:10.1093/cid/cix1085. 29462280 PMC6018983

[6] Seekatz AM, Theriot CM, Molloy CT, Wozniak KL, Bergin IL, Young VB. 2015. Fecal microbiota transplantation eliminates *Clostridium difficile* in a murine model of relapsing disease. Infect Immun 83:3838–3846. doi:10.1128/IAI.00459-15. 26169276 PMC4567621

[7] Giannasca PJ, Zhang ZX, Lei WD, Boden JA, Giel MA, Monath TP, Thomas WD, Jr., 1999. Serum antitoxin antibodies mediate systemic and mucosal protection from *Clostridium difficil*e disease in hamsters. Infect Immun 67:527–538. doi:10.1128/IAI.67.2.527-538.1999. 9916055 PMC96351

[8] Johnson S, Gerding DN. 2019. Bezlotoxumab. Clin Infect Dis 68:699–704. doi:10.1093/cid/ciy577. 30020417

[9] Gupta SB, Mehta V, Dubberke ER, Zhao X, Dorr MB, Guris D, Molrine D, Leney M, Miller M, Dupin M, Mast TC. 2016. Antibodies to toxin B are protective against *Clostridium difficile* infection recurrence. Clin Infect Dis 63:730–734. doi:10.1093/cid/ciw364. 27365387

[10] Gerding DN, Meyer T, Lee C, Cohen SH, Murthy UK, Poirier A, Van Schooneveld TC, Pardi DS, Ramos A, Barron MA, Chen H, Villano S. 2015. Administration of spores of nontoxigenic *Clostridium difficile* strain M3 for prevention of recurrent *C. difficile* infection: a randomized clinical trial. JAMA 313:1719–1727. doi:10.1001/jama.2015.3725. 25942722

[11] Gerding DN, Sambol SP, Johnson S. 2018. Non-toxigenic *Clostridioides* (formerly *Clostridium*) difficile for prevention of *C. difficile* infection: from bench to bedside back to bench and back to bedside. Front Microbiol 9:339. doi:10.3389/fmicb.2018.01700. 30093897 PMC6070627

[12] Wilson KH, Perini F. 1988. Role of competition for nutrients in suppression of *Clostridium difficile* by the colonic microflora. Infect Immun 56:2610–2614. doi:10.1128/IAI.56.10.2610-2614.1988. 3417352 PMC259619

[13] Reeves AE, Theriot CM, Bergin IL, Huffnagle GB, Schloss PD, Young VB. 2011. The interplay between microbiome dynamics and pathogen dynamics in a murine model of *Clostridium difficile* infection. Gut Microbes 2:145–158. doi:10.4161/gmic.2.3.16333. 21804357 PMC3225775

[14] Kelly ML, Ng YK, Cartman ST, Collery MM, Cockayne A, Minton NP. 2016. Improving the reproducibility of the NAP1/B1/027 epidemic strain R20291 in the hamster model of infection. Anaerobe 39:51–53. doi:10.1016/j.anaerobe.2016.02.011. 26946361 PMC4879870

[15] Leslie JL, Vendrov KC, Jenior ML, Young VB. 2019. The gut microbiota is associated with clearance of *Clostridium difficile* infection independent of adaptive immunity. mSphere 4:e00698-18. doi:10.1128/mSphereDirect.00698-18. 30700514 PMC6354811

[16] Mombaerts P, Iacomini J, Johnson RS, Herrup K, Tonegawa S, Papaioannou VE. 1992. RAG-1-deficient mice have no mature B and T lymphocytes. Cell 68:869–877. doi:10.1016/0092-8674(92)90030-G. 1547488

[17] Jarchum I, Liu M, Lipuma L, Pamer EG. 2011. Toll-like receptor 5 stimulation protects mice from acute *Clostridium difficile* colitis. Infect Immun 79:1498–1503. doi:10.1128/IAI.01196-10. 21245274 PMC3067529

[18] Jarchum I, Liu M, Shi C, Equinda M, Pamer EG. 2012. Critical role for MyD88-mediated neutrophil recruitment during *Clostridium difficile* colitis. Infect Immun 80:2989–2996. doi:10.1128/IAI.00448-12. 22689818 PMC3418725

[19] Sang L-X, Chang B, Wang B-Y, Liu W-X, Jiang M. 2015. Live and heat-killed probiotic: effects on chronic experimental colitis induced by dextran sulfate sodium (DSS) in rats. Int J Clin Exp Med 8:20072–20078. 26884919 PMC4723764

[20] Chung I-C, OuYang C-N, Yuan S-N, Lin H-C, Huang K-Y, Wu P-S, Liu C-Y, Tsai K-J, Loi L-K, Chen Y-J, Chung A-K, Ojcius DM, Chang Y-S, Chen L-C. 2019. Pretreatment with a heat-killed probiotic modulates the NLRP3 inflammasome and attenuates colitis-associated colorectal cancer in mice. Nutrients 11:516. doi:10.3390/nu11030516. PMC6471765 30823406

[21] Behroozian AA, Chludzinski JP, Lo ES, Ewing SA, Waslawski S, Newton DW, Young VB, Aronoff DM, Walk ST. 2013. Detection of mixed populations of *Clostridium difficil*e from symptomatic patients using capillary-based polymerase chain reaction ribotyping. Infect Control Hosp Epidemiol 34:961–966. doi:10.1086/671728. 23917911 PMC4016961

[22] Seekatz AM, Wolfrum E, DeWald CM, Putler RKB, Vendrov KC, Rao K, Young VB. 2018. Presence of multiple *Clostridium difficile* strains at primary infection is associated with development of recurrent disease. Anaerobe 53:74–81. doi:10.1016/j.anaerobe.2018.05.017. 29859301 PMC6274632

[23] Wilson KH, Sheagren JN. 1983. Antagonism of toxigenic *Clostridium difficile* by nontoxigenic *C. difficile*. J Infect Dis 147:733–736. doi:10.1093/infdis/147.4.733. 6842009

[24] Szczesny A, Martirosian G, Cohen S, Silva J, Jr., 2005. Co-infection of hamsters with toxin A or toxin B-deficient *Clostridium difficile* strains. Pol J Microbiol 54:301–304. 16599301

[25] Etienne-Mesmin L, Chassaing B, Adekunle O, Mattei LM, Bushman FD, Gewirtz AT. 2018. Toxin-positive *Clostridium difficile* latently infect mouse colonies and protect against highly pathogenic *C. difficile*. Gut 67:860–871. doi:10.1136/gutjnl-2016-313510. 28219893 PMC5941301

[26] Borriello SP, Barclay FE. 1985. Protection of hamsters against *Clostridium difficile* ileocaecitis by prior colonisation with non-pathogenic strains. J Med Microbiol 19:339–350. doi:10.1099/00222615-19-3-339. 4009689

[27] Jenior ML, Leslie JL, Young VB, Schloss PD. 2017. *Clostridium difficile* colonizes alternative nutrient niches during infection across distinct murine gut microbiomes. mSystems 2:e00063-17. doi:10.1128/mSystems.00063-17. 28761936 PMC5527303

[28] Bouillaut L, Self WT, Sonenshein AL. 2013. Proline-dependent regulation of *Clostridium difficile* Stickland metabolism. J Bacteriol 195:844–854. doi:10.1128/JB.01492-12. 23222730 PMC3562115

[29] Neumann-Schaal M, Hofmann JD, Will SE, Schomburg D. 2015. Time-resolved amino acid uptake of *Clostridium difficile* 630Δerm and concomitant fermentation product and toxin formation. BMC Microbiol 15:1–12. doi:10.1186/s12866-015-0614-2. 26680234 PMC4683695

[30] Battaglioli EJ, Hale VL, Chen J, Jeraldo P, Ruiz-Mojica C, Schmidt BA, Rekdal VM, Till LM, Huq L, Smits SA, Moor WJ, Jones-Hall Y, Smyrk T, Khanna S, Pardi DS, Grover M, Patel R, Chia N, Nelson H, Sonnenburg JL, Farrugia G, Kashyap PC. 2018. *Clostridioides difficile* uses amino acids associated with gut microbial dysbiosis in a subset of patients with diarrhea. Sci Transl Med 10:eaam7019. doi:10.1126/scitranslmed.aam7019. 30355801 PMC6537101

[31] Sorg JA, Sonenshein AL. 2008. Bile acids and glycine as cogerminants for *Clostridium difficile* spores. J Bacteriol 190:2505–2412. doi:10.1128/JB.01765-07. 18245298 PMC2293200

[32] Lawley TD, Clare S, Walker AW, Goulding D, Stabler RA, Croucher N, Mastroeni P, Scott P, Raisen C, Mottram L, Fairweather NF, Wren BW, Parkhill J, Dougan G. 2009. Antibiotic treatment of *Clostridium difficile* carrier mice triggers a supershedder state, spore-mediated transmission, and severe disease in immunocompromised hosts. Infect Immun 77:3661–3669. doi:10.1128/IAI.00558-09. 19564382 PMC2737984

[33] Shrestha R, Sorg JA. 2018. Hierarchical recognition of amino acid co-germinants during *Clostridioides difficile* spore germination. Anaerobe 49:41–47. doi:10.1016/j.anaerobe.2017.12.001. 29221987 PMC5844826

[34] Buffie CG, Bucci V, Stein RR, McKenney PT, Ling L, Gobourne A, No D, Liu H, Kinnebrew M, Viale A, Littmann E, van den Brink MRM, Jenq RR, Taur Y, Sander C, Cross JR, Toussaint NC, Xavier JB, Pamer EG. 2015. Precision microbiome reconstitution restores bile acid mediated resistance to *Clostridium difficile*. Nature 517:205–208. doi:10.1038/nature13828. 25337874 PMC4354891

[35] Studer N, Desharnais L, Beutler M, Brugiroux S, Terrazos MA, Menin L, Schürch CM, McCoy KD, Kuehne SA, Minton NP, Stecher B, Bernier-Latmani R, Hapfelmeier S. 2016. Functional intestinal bile acid 7α-dehydroxylation by associated with protection from infection in a gnotobiotic mouse model. Front Cell Infect Microbiol 6:191. doi:10.3389/fcimb.2016.00191. 28066726 PMC5168579

[36] Nagaro KJ, Phillips ST, Cheknis AK, Sambol SP, Zukowski WE, Johnson S, Gerding DN. 2013. Nontoxigenic *Clostridium difficile* protects hamsters against challenge with historic and epidemic strains of toxigenic BI/NAP1/027 *C. difficile*. Antimicrob Agents Chemother 57:5266–5270. doi:10.1128/AAC.00580-13. 23939887 PMC3811292

[37] Lanis JM, Barua S, Ballard JD. 2010. Variations in TcdB activity and the hypervirulence of emerging strains of *Clostridium difficile*. PLoS Pathog 6:e100106. doi:10.1371/journal.ppat.1001061. PMC2924371 20808849

[38] Fabich AJ, Jones SA, Chowdhury FZ, Cernosek A, Anderson A, Smalley D, McHargue JW, Hightower GA, Smith JT, Autieri SM, Leatham MP, Lins JJ, Allen RL, Laux DC, Cohen PS, Conway T. 2008. Comparison of carbon nutrition for pathogenic and commensal *Escherichia coli* strains in the mouse intestine. Infect Immun 76(3):1143–1152. doi:10.1128/IAI.01386-07. 18180286 PMC2258830

[39] Schinner SAC, Mokszycki ME, Adediran J, Leatham-Jensen M, Conway T, Cohen PS. 2015. *Escherichia coli* EDL933 requires gluconeogenic nutrients to successfully colonize the intestines of streptomycin-treated mice precolonized with *E. coli* Nissle 1917. Infect Immun 83:1983–1991. doi:10.1128/IAI.02943-14. 25733524 PMC4399065

[40] Hardin G. 1960. The competitive exclusion principle. Science 131:1292–1297. doi:10.1126/science.131.3409.1292. 14399717

[41] Connell JH. 1990. Apparent versus “real” competition in plants, p 9–26. *In* Grace JB, Tilman D (ed), Perspectives on plant competition. Academic Press, San Diego, CA.

[42] Thanissery R, Winston JA, Theriot CM. 2017. Inhibition of spore germination, growth, and toxin activity of clinically relevant *C. difficile* strains by gut microbiota derived secondary bile acids. Anaerobe 45:86–100. doi:10.1016/j.anaerobe.2017.03.004. 28279860 PMC5466893

[43] Warren CA, van Opstal E, Ballard TE, Kennedy A, Wang X, Riggins M, Olekhnovich I, Warthan M, Kolling GL, Guerrant RL, Macdonald TL, Hoffman PS. 2012. Amixicile, a novel inhibitor of pyruvate:ferredoxin oxidoreductase, shows efficacy against *Clostridium difficile* in a mouse infection model. Antimicrob Agents Chemother 56:4103–4111. doi:10.1128/AAC.00360-12. 22585229 PMC3421617

[44] Theriot CM, Koumpouras CC, Carlson PE, Bergin II, Aronoff DM, Young VB. 2011. Cefoperazone-treated mice as an experimental platform to assess differential virulence of *Clostridium difficile* strains. Gut Microbes 2:326–334. doi:10.4161/gmic.19142. 22198617 PMC3337121

[45] Untergasser A, Cutcutache I, Koressaar T, Ye J, Faircloth BC, Remm M, Rozen SG. 2012. Primer3—new capabilities and interfaces. Nucleic Acids Res 40:e115. doi:10.1093/nar/gks596. 22730293 PMC3424584

[46] Theriot CM, Bowman AA, Young VB. 2016. Antibiotic-induced alterations of the gut microbiota alter secondary bile acid production and allow for *Clostridium difficile* spore germination and outgrowth in the large intestine. mSphere 1:e00045-15. doi:10.1128/mSphere.00045-15. PMC4863611 27239562

[47] Sas KM, Kayampilly P, Byun J, Nair V, Hinder LM, Hur J, Zhang H, Lin C, Qi NR, Michailidis G, Groop P-H, Nelson RG, Darshi M, Sharma K, Schelling JR, Sedor JR, Pop-Busui R, Weinberg JM, Soleimanpour SA, Abcouwer SF, Gardner TW, Burant CF, Feldman EL, Kretzler M, Brosius FC, III, Pennathur S. 2016. Tissue-specific metabolic reprogramming drives nutrient flux in diabetic complications. JCI Insight 1:e86976. doi:10.1172/jci.insight.86976. 27699244 PMC5033761

[48] Kozich JJ, Westcott SL, Baxter NT, Highlander SK, Schloss PD. 2013. Development of a dual-index sequencing strategy and curation pipeline for analyzing amplicon sequence data on the MiSeq Illumina sequencing platform. Appl Environ Microbiol 79:5112–5120. doi:10.1128/AEM.01043-13. 23793624 PMC3753973

[49] Schloss PD, Westcott SL, Ryabin T, Hall JR, Hartmann M, Hollister EB, Lesniewski RA, Oakley BB, Parks DH, Robinson CJ, Sahl JW, Stres B, Thallinger GG, Van Horn DJ, Weber CF. 2009. Introducing mothur: open-source, platform-independent, community-supported software for describing and comparing microbial communities. Appl Environ Microbiol 75:7537–7541. doi:10.1128/AEM.01541-09. 19801464 PMC2786419

[50] Quast C, Pruesse E, Yilmaz P, Gerken J, Schweer T, Yarza P, Peplies J, Glöckner FO. 2013. The SILVA ribosomal RNA gene database project: improved data processing and web-based tools. Nucleic Acids Res 41:D590–6. doi:10.1093/nar/gks1219. 23193283 PMC3531112

[51] Edgar RC, Haas BJ, Clemente JC, Quince C, Knight R. 2011. UCHIME improves sensitivity and speed of chimera detection. Bioinformatics 27:2194–2200. doi:10.1093/bioinformatics/btr381. 21700674 PMC3150044

[52] Wang Q, Garrity GM, Tiedje JM, Cole JR. 2007. Naive Bayesian classifier for rapid assignment of rRNA sequences into the new bacterial taxonomy. Appl Environ Microbiol 73:5261–5267. doi:10.1128/AEM.00062-07. 17586664 PMC1950982

[53] Westcott SL, Schloss PD. 2017. OptiClust, an improved method for assigning amplicon-based sequence data to operational taxonomic units. mSphere 2:e00073-17. doi:10.1128/mSphereDirect.00073-17. 28289728 PMC5343174

[54] Tierney L. 2012. The R statistical computing environment. *In* Feigelson E, Babu G (eds) Statistical challenges in modern astronomy V. Lecture notes in statistics, vol 902. Springer, New York, NY.

